# Hydrogel Versus Alternative Vehicles for (Trans)dermal Delivery of Propranolol Hydrochloride—In Vitro and Ex Vivo Studies

**DOI:** 10.3390/gels12010010

**Published:** 2025-12-23

**Authors:** Nataša Bubić Pajić, Milica Kaurin, Adrijana Klepić, Darija Knežević Ratković, Aneta Stojmenovski, Veljko Krstonošić, Ranko Škrbic

**Affiliations:** 1 Department of Pharmacy, Faculty of Medicine, University of Banja Luka, Save Mrkalja 14, 78000 Banja Luka, Bosnia and Herzegovina; darija.knezevic.ratkovic@med.unibl.org; 2 Berlin-Chemie Menarini AG, Hasana Brkica 2/II, 71000 Sarajevo, Bosnia and Herzegovina; kaurinmilica@gmail.com; 3 ZU Apoteke “B Pharm”, Kulska Obala Bb, 79220 Novi Grad, Bosnia and Herzegovina; klepicadrijana@yahoo.com; 4 Centre for Biomedical Research, Faculty of Medicine, University of Banja Luka, Save Mrkalja 16, 78000 Banja Luka, Bosnia and Herzegovina; aneta.stojmenovski@med.unibl.org; 5 Department of Pharmacy, Faculty of Medicine, University of Novi Sad, Hajduk Veljkova 3, 21000 Novi Sad, Serbia; veljko.krstonosic@mf.uns.ac.rs; 6 Department of Pharmacology, Toxicology and Clinical Pharmacology, Faculty of Medicine, University of Banja Luka, Save Mrkalja 16, 78000 Banja Luka, Bosnia and Herzegovina; ranko.skrbic@med.unibl.org; 7 Academy of Sciences and Arts of the Republic of Srpska, Bana dr Todora Lazarevića 1, 78000 Banja Luka, Bosnia and Herzegovina; 8 Department of Pathologic Physiology, I.M. Sechenov First Moscow State Medical University, Moscow 119435, Russia

**Keywords:** propranolol hydrochloride, intradermal, transdermal, semisolid, hydrogel, microemulsion, microneedles

## Abstract

The development of advanced macromolecular systems with tailored structural and functional properties is a key objective in modern materials science, particularly for biomedical applications such as targeted drug delivery. In this study, hydrogel (HG), a polymer-based formulation, was investigated as a functional carrier for the enhanced intradermal and transdermal delivery of propranolol hydrochloride (PRO-HCl), a highly water-soluble model compound, and its potential was compared to other vehicles easily obtained by pharmacists: ointment (OM), liposomal cream (LCR), and microemulsion (ME). The formulations were characterized by their physicochemical and rheological characteristics, and evaluated in vitro and ex vivo using vertical diffusion cells equipped with synthetic membranes, intact porcine skin, and skin pretreated with solid microneedles (MNs). The HG formulation exhibited superior release performance (2396.85 ± 48.18 μg/cm^2^) and the highest intradermal drug deposition (19.87 ± 4.12 μg/cm^2^), while its combination with MNs significantly enhanced transdermal permeation (*p* = 0.0017). In contrast, the synergistic effect of MNs and ME led to a pronounced increase in drug accumulation within the skin (up to 60.3-fold). These findings highlight the crucial role of matrix composition and properties in modulating molecular transport through biological barriers. The study demonstrates that polymeric HGs represent versatile, functional materials with tunable structural and mechanical features, suitable for controlled release and potential systemic delivery applications.

## 1. Introduction

**Figure 1:**
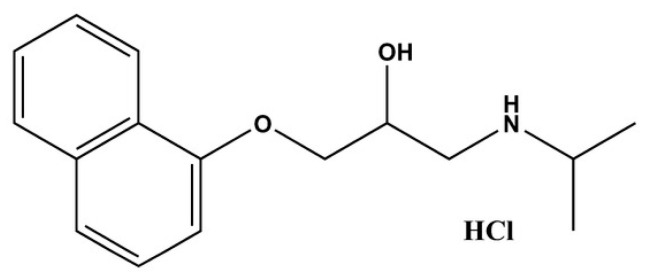
Chemical structure of PRO-HCl (Created with ChemDraw Ultra 7.0).

Several drugs manage both systemic and skin diseases. One of those drugs is PRO-HCl (Figure 1). PRO-HCl belongs to the class of non-selective β-blocking agents. It is indicated for various cardiovascular conditions including hypertension, arrhythmias, prophylaxis of myocardial infarction and angina. It is also approved for the prophylaxis of migraine, a common central nervous system disease [1,2]. In dermatology, PRO-HCl has been FDA-approved as the first-line treatment for infantile hemangioma since 2008, one of the most common benign blood vessel skin tumors in infants [3]. PRO-HCl exerts multiple mechanisms of action in infantile hemangiomas, including vasoconstriction, antiangiogenesis, inhibition of cell growth and induction of apoptosis [4,5].

Regardless of the aforementioned indications, PRO-HCl is most commonly used in oral solid (e.g., tablets) and liquid dosage forms (e.g., solutions). Intravenous preparations are also available, but exclusively for hospitalized patients [1]. However, oral PRO-HCl therapy can cause severe systemic side effects including alterations in sleep patterns, gastrointestinal symptoms, acrocyanosis, hypotension, bronchospasm, bradycardia, and hypoglycemia [6]. Additionally, to maintain a therapeutic effect, PRO-HCl’s 4 h half-life requires frequent dosing. Despite being readily absorbed from the gastrointestinal tract, PRO-HCl undergoes extensive first-pass metabolism, resulting in an oral bioavailability of less than 23% [1]. Therefore, transdermal delivery of PRO-HCl for cardiovascular diseases offers several advantages, including improved systemic bioavailability by avoiding first-pass metabolism, reduced dosing frequency, and improved patient compliance [7,8,9].

Topical PRO-HCl application is also a beneficial approach for the treatment of infantile hemangiomas, providing higher local drug concentrations at the site of action, reduced systemic exposure, and consequently fewer side effects than the oral route [6,10]. Furthermore, administering topical preparations to infants is easier compared to oral dosage forms [4].

Despite these benefits, PRO-HCl is not commercially available as a topical preparation specifically optimized for the skin or as a transdermal formulation for cardiovascular indications. The only alternative represents the extemporaneous preparation of suitable formulations by pharmacists [10]. For these purposes, PRO-HCl is usually incorporated into OMs, creams, or gels, which can be prepared according to the official pharmaceutical formularies or employed as ready-to-use vehicles [8,10]. It is also not unusual for the drug to be loaded into these vehicles in powder form obtained after crushing solid dosage forms (e.g., tablets) [8]. It is well known that the choice of the vehicle may significantly influence drug permeation across the skin due to the distinct drug release patterns and interactions with skin tissues [8,10]. Nevertheless, many of these formulations remain insufficiently characterized in terms of drug release, permeation, and clinical performance.

However, the major challenge in intra- and transdermal drug delivery is to overcome the significant barrier properties of the skin, particularly its superficial horny layer, the stratum corneum (SC) This outermost skin layer is layer 10–15 µm thick and composed of corneocytes embedded in a lipid matrix of ceramides, cholesterol, and fatty acids, providing the primary resistance to permeation, permitting mainly small, lipophilic molecules to diffuse through [11]. Hence, this highly ordered lamellar structure tightly regulates permeability and minimizes transepidermal water loss, making the SC the main obstacle for both intradermal and transdermal drug delivery [12]. In addition to these structural constraints, skin permeation is further modulated by physiological factors (such as temperature, hydration, and anatomical site), the drug’s physicochemical properties (including lipophilicity, molecular size, and diffusion/partition coefficients), and formulation-dependent parameters such as dosage form, pH, and applied concentration [13]. Drug transport can occur via intercellular, transcellular, or appendageal pathways, yet drugs often require additional enhancement strategies to overcome the SC and tight junctions located in deeper epidermal layers [11]. Therefore, the effective (trans)dermal drug delivery is limited to a small number of active pharmaceutical agents with appropriate physicochemical properties (e.g., small, lipophilic and potent molecules). PRO-HCl does not entirely fit these properties due to its high hydrophilicity (BCS class I, logP = 1.16 ± 0.57 and pKa 9.50 ± 0.15), restricting its passive diffusion into/through the deeper skin layers [6,14]. Namely, PRO-HCl permeation across the SC requires the consecutive drug partitioning between hydrophilic domain (keratin) and hydrophobic lipid part of the SC. But in the case of hydrophilic drugs, the latter lipid bilayer environment is difficult to overcome [15].

Another challenge associated with topical drug administration lies in achieving selective intradermal or transdermal drug delivery. To target skin diseases and alleviate symptoms of thereof, local concentrations of the drug within the skin tissues are desired, ideally without systemic drug bioavailability. However, despite being unwanted in this context, some degree of systemic absorption it often unavoidable. In contrast to intradermal drug transport, selective transdermal drug delivery aims for a therapeutically relevant level of a drug in the systemic circulation, with minimal local drug deposition. In this latter case, some local drug presence is, by nature, inevitable [15].

As already mentioned, the main challenge in developing a dermal or transdermal PRO-HCl delivery system arises from its hydrophilic nature and the lipophilic barrier function of the SC. To overcome this limitation, various formulations strategies, including the use of HGs, OMs, MEs, and physical enhancement techniques such as MNs, have been explored. However, studies specifically addressing PRO-HCl skin retention are very scarce, most experimental works have assessed drug permeation, considering it as the primary indication of the drug skin delivery [16]. Due to their high water content, biocompatibility, ease of preparation, and favorable sensory characteristics, among various pharmaceutical vehicles, HGs have emerged as particularly promising and widely used as functional biomaterials for tissue repair and drug/gene delivery [17]. The three-dimensional polymeric network of HGs enables the incorporation of hydrophilic or amphiphilic drug molecules, maintains skin hydration, and promotes intimate contact with the SC, thereby enhancing both comfort and efficacy upon application. These attributes distinguish HGs from other conventional semisolid or colloidal carriers, such as OMs, creams, or emulsions, which often rely on occlusive effects or surfactant-mediated penetration enhancement [18,19,20]. Recent studies also highlighted the importance of HG structural features for controlling payload release and tissue interactions implicated in enhanced wound-healing and mucosal repair applications [21,22,23]. In this context, HGs stand out as pharmaceutically accessible formulations that can be easily prepared in compounding settings while offering potential for optimized dermal delivery. Despite these advancements, most prior studies focused on single formulation platforms or compared similar systems under varying conditions, leaving a lack of systematic evaluation across conventional and nanostructured vehicles.

**Figure 2:**
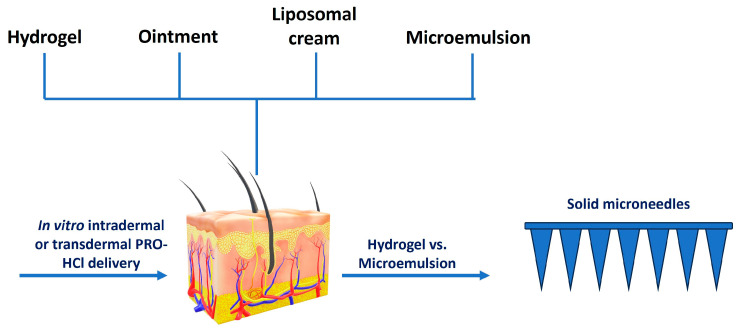
Scheme illustrating the study design.

To enable a systematic comparison of different topical delivery systems, this study evaluated the performance of a HG for intradermal delivery, particularly suitable for infantile hemangioma, and for transdermal delivery, relevant to cardiovascular indications. The HG was compared under identical experimental conditions with representative topical vehicles: a conventional pharmacopeial-quality OM, a commercially available ready-to-use lipid-based colloidal nanocarrier (LCR), and a submicron colloidal nanocarrier (ME) (Figure 2). Special attention was given to the relationship between vehicle composition, physicochemical characteristics, and drug transport behavior. To further explore the potential for enhancing systemic delivery, selected formulations were combined with MN pretreatment to assess possible synergistic effects on PRO-HCl penetration (Figure 2).

Therefore, the aim of this study was to determine how vehicle composition and MN pretreatment influence the intradermal and transdermal disposition of PRO-HCl, and to identify delivery strategies that optimize its distribution within and across the skin.

## 2. Results and Discussion

### 2.1. Selection of Formulations

Because existing extemporaneous PRO-HCl formulations are not adequately characterized in terms of drug release, skin penetration, and overall (trans)dermal performance, a systematic comparative evaluation of different vehicles is needed. Given this gap, the present study primarily focused on the evaluation of a pharmacopeial carmellose-based HG as a vehicle for (trans)dermal delivery of PRO-HCl. HGs offer several intrinsic advantages that make them particularly suitable for this purpose. Because physical or chemical cross-links are present in HG matrices, these networks have the capacity to imbibe a large amount of water and remain insoluble afterwards [24]. Due to the relatively high water content, HGs provide skin moisturization, enhance elasticity and adhesion properties, and offer a more pleasant sensation upon application to the skin, making them a beneficial alternative to OMs and creams [19]. Moreover, the rheological tunability of HGs allows for fine control over spreadability, mechanical resilience, and drug release kinetics, representing properties that are directly linked to therapeutic performance and patient acceptability [18,19,20]. In addition to this, pharmacopeial HGs are recognized as biocompatible carriers due to their high water content and minimal interaction with skin tissue. Their long-standing use in magistral dermatological preparations further supports their safety for topical application.

To provide a comprehensive comparison and establish a reference framework, three other vehicle types were selected: an OM representing a traditional occlusive matrix, a LCR as a vesicular system offering both hydration and penetration enhancement, and a ME as a nanostructured, thermodynamically stable carrier. Each of these alternatives exhibits specific advantages, yet differs fundamentally in composition, internal structure, and rheological behavior. While OMs ensure prolonged contact with the skin via occlusion [8], HGs achieve sustained delivery through controlled diffusion within their hydrated polymeric network. LCRs and MEs, on the other hand, utilize dispersed nanophases to modulate skin permeation [25,26,27,28,29]. Here, the LCR used was commercial ready-to-use base Pentravan^®^. The ME formulation investigated in this study was selected from a pseudo-ternary phase diagram (Supplementary Materials Figure S1). The components used for this diagram were selected based on their satisfactory drug solubilization capacity (Supplementary Materials Table S1), as determined by the shake-flask method (Supplementary Materials). Based on earlier findings [14,29,30,31,32], the O/W ME system containing 60% (*w*/*w*) of water was considered the most suitable vehicle for exploring topical delivery of PRO-HCl in this study.

To further enhance the delivery potential, the selected systems were also investigated in combination with MN pretreatment. “Poke and patch” approach is relatively simple method that allows enhanced drug transport from a topical formulation into/through the skin upon insertion and subsequent removal of solid MNs [33]. When combined with HGs, this strategy offers a particularly synergistic platform, i.e., pairing mechanical barrier disruption with diffusion-controlled release from a hydrated matrix. Despite this potential, the influence of MN-assisted delivery on PRO-HCl transport from HGs has not been systematically studied before. Generally, improvements in MNs-mediated (trans)dermal delivery of hydrophilic PRO-HCl are scarcely explored in the relevant literature [6,14].

### 2.2. Formulations Characterization

The fundamental physicochemical characteristics of the prepared formulations were examined to elucidate their potential impact on drug release and skin permeation behavior. All systems were macroscopically homogeneous, with PRO-HCl uniformly distributed throughout the matrices. In the case of the HG, the drug was completely dissolved within the aqueous polymeric network, consistent with its high aqueous solubility, ensuring molecular-level dispersion and uniform distribution within the gel matrix. This homogeneity is expected to promote reproducible diffusion-controlled release and enhance formulation stability.

No traces of undissolved drug were noticeable in the LCR and MEs samples, which was expected due to their high content of water. In contrast, in the OM, PRO-HCl remained largely suspended as undissolved particles due to the hydrophobic nature of the base. The particle size of PRO-HCl dispersed in OM ranged broadly from 2.61 to 169.54 µm, which was likely a consequence of using the drug substance as received, without additional mechanical size reduction steps, or due to the partial solubilization of the drug in the OM base.

Please note that the characterization approach differed among formulations depending on their physicochemical nature. For the HG and LCR, PRO-HCl is molecularly dissolved within the aqueous phase, and therefore no discrete colloidal particles are formed. Hence, particle size, particle size distribution, and zeta potential measurements are not applicable to these systems. In contrast, droplet size and PDI were determined for the ME. However, for the ointment OM, only the size range of PRO-HCl dispersed particles could be determined, as the available software did not support PDI analysis.

**Figure 3:**
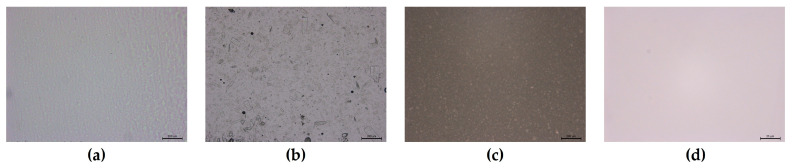
Representative micrographs of the investigated semisolid formulations: (**a**) hydrogel (HG); (**b**) ointment (OM); (**c**) liposomal cream (LCR); (**d**) ME. Scale bar 200 µm (**a**–**c**) and 25 µm (**d**).

Acceptable drug content uniformity and stability were confirmed upon 1 month of storage at room temperature for each sample (Table 1). However, somewhat lower drug content within the OM sample might be due to either incomplete extraction or non-uniform distribution of PRO-HCl (Figure 3).

The pH values were slightly acidic, ranging from 5.19 to 7.08 (Table 1). Given that pKa of PRO-HCl is 9.5, greater fraction of the drug exists in its unionized form within the aqueous phase of formulations when their pH values are closer to this pKa. Consequently, the skin permeation potential of the investigated formulations can be presumed depending on the ratio of unionized to ionized drug amount present [34,35].

The electrical conductivity of the blank ME was 196.53 ± 5.08 µS/cm, suggesting an O/W system was formed. After PRO-HCl solubilization in this sample, the electrical conductivity rose dramatically (1126.33 ± 17.62 µS/cm). This was expected due to the drug’s ionization in the aqueous environment, which enabled chloride anions to pass through water channels. The average size of the oil droplets dispersed in water was 153.67 ± 54.73 nm with a polydispersity index (PDI) of 0.237 ± 0.023, suggesting a unimodal size distribution.

**Figure 4:**
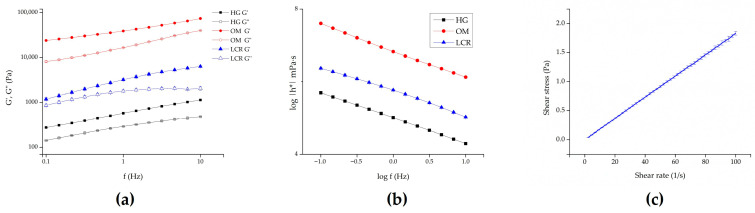
Rheological behavior of the investigated samples: (**a**) storage (G’) and loss (G’’) modulus of semisolid formulations as a function of frequency; (**b**) complex viscosity of semisolid samples as a function of oscillation frequency; and (**c**) ME. HG—hydrogel; OM—ointment; LCR—liposomal cream.

Figure 4 presents the rheological behavior of the tested formulations. The semisolid formulations investigated in this study exhibited more complex rheological properties, as confirmed by oscillatory rheological measurements. As Figure 4a illustrates, for each semisolid sample, G’ dominates over G’’ across the entire range of frequencies, indicating that the formulations behave as viscoelastic solids. Additionally, samples with higher G’ values also demonstrated higher complex viscosity (Figure 4b). The complex viscosity is represented by the equation η* = G*/ω, where G* = τ_A_/γ_A_ (τ_A_ is the shear stress amplitude and γ_A_ is the strain amplitude). It is evident that complex viscosities for all samples decreased as frequency increased. Assuming the Cox-Mertz rule can be applied to these systems, it can be concluded that all systems behave as non-Newtonian, pseudoplastic materials [36,37]. Accordingly, HG exhibited the lowest complex viscosity compared to the other two samples, suggesting different texture characteristics and potentially easier spreading of the formulation and the incorporated drug on the skin surface. Hence, the gel formulation exhibited adequate viscosity and spreadability, ensuring uniform application and retention on the skin surface throughout the penetration/permeation experiments. No macroscopic changes, such as phase separation or drying, were observed, confirming its suitability for sustained drug delivery. In addition to this, drug delivery can also be affected by the rheological properties of a vehicle. This occurs either by influencing the drug diffusion process through the vehicle itself or by causing enhanced skin hydration, which modulates its permeability [34]. Conversely, in the case of the ME, we observed Newtonian flow behavior with low viscosity (18.29 ± 0.33 mPa·s) (Figure 4c), confirming that the developed system aligned with typical MEs’ characteristics, lacking the presence of liquid crystalline structures.

### 2.3. In Vitro Drug Release Studies

Adequate in vitro drug release studies provide important information on the process of drug liberation from topical formulations, including the underlying mechanisms and factors affecting drug release. These studies indirectly assess a formulation’s bioavailability and therapeutic efficacy [38]. In the current study, we investigated the HG sample and compared it with diverse semisolid and ME samples using artificial cellulose dialysis membranes. Our goal was to detect potential differences in their drug release patterns.

Dialysis membranes are established as effective in ensuring that only the released drug reaches the receptor compartment, especially when validating that the formulation itself is retained. However, in order to select dialysis membrane with appropriate characteristics, the cut-off point should be considered. As previously recommended, the molecular weight cut-off about 100 times the molecular weight of the drug [39]. However, the commonly used molecular weight cut-off is 12–70 times greater than the molecular weight of the tested substance [39,40,41,42], which is fulfilled in our study.

**Figure 5:**
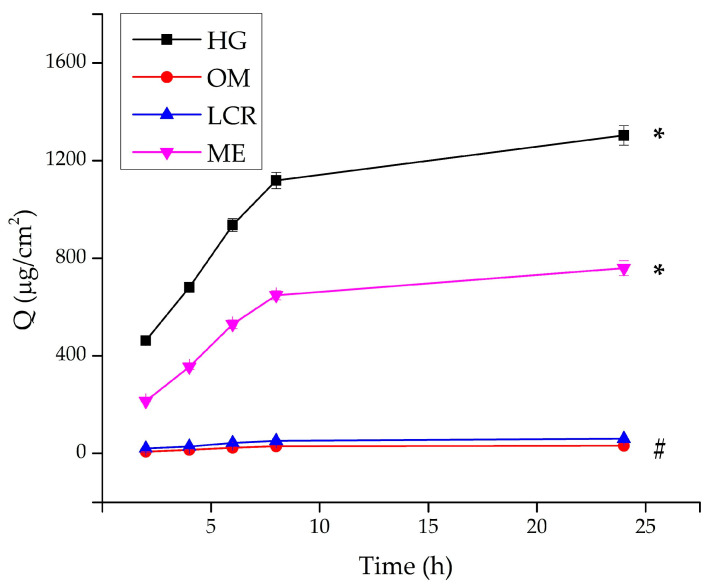
In vitro release of PRO-HCl from the investigated formulations determined using the synthetic cellulose dialysis membrane, under *infinite* dose conditions (mean ± SD, *n* = 3). * Statistically significant change (*p* < 0.05) at the end of the experiment compared to all other samples; # statistically significant change (*p* < 0.05) at the end of the experiment compared to HG and ME. HG-hydrogel; OM—ointment; LCR—liposomal cream; ME—microemulsion.

Figure 5 and Table 2 present the cumulative amount of PRO-HCl (µg/cm^2^) released from the tested formulations over 24 h.

The release study revealed pronounced differences in the drug release profiles among the tested formulations. The HG, composed of carmellose sodium, exhibited by far the highest PRO-HCl release rate, reaching 2396.86 ± 59.80 μg/cm^2^ after 24 h, corresponding to approximately 48% of the total incorporated drug. This result clearly demonstrates the efficiency of the hydrated polymeric matrix to facilitate diffusion of the hydrophilic drug through its porous network. The high water content, uniform drug solubilization, and viscoelastic gel structure jointly contributed to continuous and sustained release behavior.

The ME formulation showed moderate release performance, reflecting its dual-phase composition and partially restricted aqueous diffusion channels. In contrast, the OM exhibited the lowest release, with only 50.41 ± 3.87 μg/cm^2^ of PRO-HCl liberated (≈1% of the total drug content) after 24 h. This limited release can be attributed to the hydrophobic character of the base, where the drug was primarily dispersed as undissolved particles. Considering the particle sizes were substantially larger than the 2.4 nm pores of the dialysis membrane, diffusion of the suspended fraction was likely hindered, further restricting drug transport.

Table 2 presents the release rates and the fraction (%) of drug released after 24 h. The release rates were determined from the slope of the curve showing the dependence of the amount of PRO-HCl released per unit area (μg/cm^2^) as a function of the square root of time, during which the release was monitored. As observed, the release rates correlated with the level of drug liberation achieved, and significant differences were found between the investigated samples.

The concentration of PRO-HCl at the target site of action after topical application directly correlates with its ability to penetrate through the SC and permeate through the epidermis. However, the drug must first diffuse through the formulation matrix and be released before it can penetrate. In this study, the highest amount of drug was released from HG, providing the fastest drug release. This result was somewhat unexpected, considering that the relevant literature suggests that faster drug release is achieved when the incorporated drug shows a lower affinity for its vehicle [34,43]. However, PRO-HCl is a hydrophilic molecule (logP octanol/PBS 7.4 = 1.16 ± 0.57) that dissolves well in water and thus has a high affinity for the HG vehicle. Nevertheless, results similar to ours have been already obtained elsewhere [10,35], suggesting that PRO-HCl release from lipophilic vehicles (OMs) was limited and negligible in compared to water-containing vehicles (creams). Moreover, a higher water percentage in formulations correlated with increase PRO-HCl [14,35]. However, despite LCR being a hydrophilic cream formulation with 62% water [44], drug release from this vehicle was significantly lower than from the other two water-containing formulations. A possible explanation for the limited release of PRO-HCl from Pentravan^®^ LCR may be the different locus of incorporation for the active substance within this sample compared to HG and ME. Namely, given PRO-HCl’s high solubility in water (Supplementary Materials Table S1) and the nature of the water-containing samples, it would be reasonable to assume that the drug is most probably solubilized in the external water phase of HG and ME. Conversely, the drug may be partitioned, at least partially, between the outer aqueous phase and the liposomal inner core of LCR, consequently affecting its release [25]. Furthermore, it cannot be neglected that the lower release of PRO-HCl from the cream formulation might originate from the possible higher diffusion of water, ethanol and glycerol from HG and ME into the acceptor medium. This would lower the drug solubility in those samples, thereby causing precipitation and increased thermodynamic activity.

By correlating the pH values of the tested formulations with the pKa of PRO-HCl (pKa = 9.5 ± 0.15) [8], it can be noticed that the highest amount of drug was released from formulations with pH values closest to the pKa value of the drug (Table 1 and Table 2). This is in a good agreement with previous studies which document PRO-HCl preparations with pH values near its pKa exhibit the most efficient permeation through human epidermis, primarily due to the drug existing in its unionized form [35]. However, the pH value of the formulation was not the sole deciding factor influencing PRO-HCl release kinetic. Other structural and compositional elements of the vehicle contribute to the thermodynamic activity of the drug. Specifically, drug release can be correlated with rheological characteristics of the formulations, at least for those with semisolid consistency. To be more precise, for these semisolid samples, drug release was inversely proportional to their apparent viscosity values (Figure 4). This trend could be anticipated, as drug diffusion is generally more difficult through a more viscous environment. While this suggests that the highest amount of PRO-HCl would be released from the least viscous formulation, the ME did not fit this pattern due to its more complex inner microstructure. It appears that drug release from the ME sample was primarily governed by its water content and pH value.

However, it should be emphasized that the following interpretations represent potential explanations for the observed differences in drug release. Given that the tested formulations differ in their internal microstructure and multiple physicochemical attributes (e.g., pH, viscosity, water content, drug solubility in the vehicle), these factors are interdependent, and their combined influence, rather than the isolated effect of a single parameter, most likely accounts for the observed trends.

Based on the experimentally obtained data, the drug release profiles were fitted to several kinetic models. In addition to conventional models such as zero-order, first-order, and Higuchi, the Korsmeyer–Peppas model was applied to analyze the drug release profile, as it allows evaluation of complex release mechanisms involving both diffusion and polymer relaxation or swelling of the HG matrix [45]. The most suitable mechanism for PRO-HCl release from the investigated samples was established by performing linear regression analysis and calculating the correlation coefficient (r^2^) values, which are presented in Table 2.

The release of PRO-HCl from HG can be best described by the Korsmeyer-Peppas. The diffusion exponent (n) value was 0.51, which falls within the range 0.5 < *n* < 1, indicating anomalous (non-Fickian) transport, in which drug diffusion occurs simultaneously with polymer chain relaxation [46]. Such a mechanism is characteristic of hydrated viscoelastic matrices, where both diffusion through aqueous channels and gradual structural rearrangement of the polymer network govern the release process. This finding supports the concept that the HG matrix based on carmellose sodium enables a dual-controlled release: diffusion-driven yet modulated by the dynamic behavior of the polymeric network. The ability of the gel to maintain structural integrity while permitting molecular mobility within the hydrated mesh contributes to its predictable and sustained release characteristics [10,25].

In contrast, the release profiles of PRO-HCl from the OM, LCR and ME formulations fitted best to Higuchi model, indicating Fickian diffusion controlled primarily by the vehicle composition and matrix structure. These systems exhibited linear release kinetics, implying a more uniform but less adaptable diffusion environment compared to the polymeric HG.

According to the findings established through the in vitro release study, HG provided the highest PRO-HCl release. Also, differences in drug delivery into/through the skin, and indirectly in the availability of the drug into skin tissues and systemic circulation, could be indicated [38]. To confirm these assumptions, an ex vivo skin penetration/permeation study was therefore conducted.

### 2.4. Ex Vivo Drug Penetration/Permeation Studies

**Figure 6:**
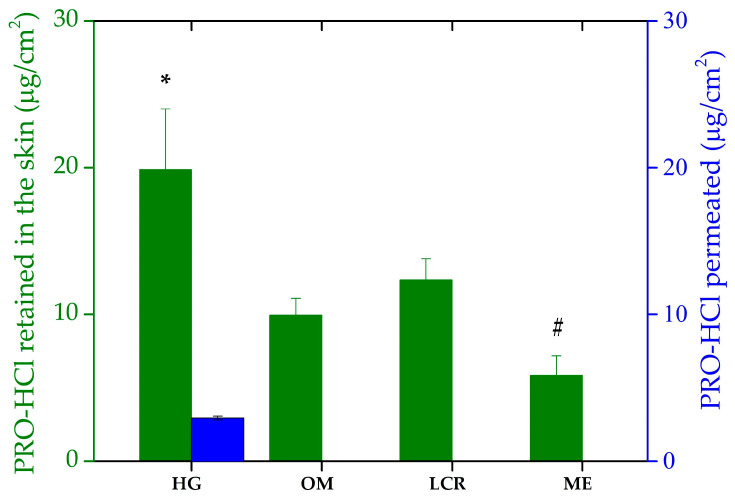
Ex vivo penetration/permeation of PRO-HCl from the investigated formulations determined across full-thickness porcine ear skin under *infinite* dose conditions (mean ± SD, *n* = 3). * Statistically significant change (*p* < 0.05) compared to OM and ME; # statistically significant change (*p* < 0.05) compared to LCR and HG. HG-hydrogel; OM—ointment; LCR—liposomal cream; ME—microemulsion.

Given that pig skin is commonly considered as a surrogate model for the initial screening of transdermal and dermal formulations, due to its close resemblance to human skin in terms of barrier properties and lipid composition [47,48,49], a penetration/permeation experiment was performed using the dorsal side of porcine ear skin. PRO-HCl delivery was assessed by measuring the amount of drug penetrated into the skin and permeated through the skin per skin surface area (μg/cm^2^) after the experiment. The values of these parameters served as a measure of the formulations’ efficacy for dermal and transdermal delivery, respectively. The results obtained from this study are presented in Figure 6 and Table 3.

Interestingly, the drug transport into the skin achieved by the investigated samples did not follow the same rank order as the in vitro drug release study. Namely, based on the amount of PRO-HCl retained in the skin, the tested formulations can be ranked as: HG > LCR > OM > ME. As Figure 6 shows, the semisolid formulations demonstrated more effective intradermal drug delivery, mirroring the trend observed in PRO-HCl release. Penetration of the drug was the highest from the HG sample, which also yielded the greatest drug release. This finding leads to a conclusion that for the semisolid vehicles, PRO-HCl uptake into the skin was limited by the drug release process. Based on the drug amount extracted from the skin per surface area, the most hydrophilic formulation (HG) provided the best skin retention (19.87 ± 4.12 µg/cm^2^). This tendency, however, was not entirely consistent with previous studies noting that applying poloxamer-based hydrophilic gels often leads to reduced drug skin concentrations and significant transdermal drug absorption [8]. Nevertheless, the higher permeability of PRO incorporated in gellan gum HG compared to the hydrophilic cream formulation with the same drug concentration supports the results of our study [4]. Furthermore, Casiraghi et al. observed the best PRO-HCl penetrating ability in the epidermis with hydrophilic cream, followed by lipophilic cream formulations and OM. They also found that PRO-HCl water and PBS solutions performed better in terms of drug transport into epidermal layer of porcine skin [35]. However, while the vehicle performances in drug release correlated well with drug penetration, they cannot fully explain all the results. Indeed, although the cumulative amount of PRO-HCl released from HG was approximately 22- and 47-fold higher than from the LCR and OM, respectively, the ratios of drug penetration from these formulations were only 1.61 and 1.99. These results indicated that while HG released a sufficient amount of drug, its partition into the SC was more hindered than that from more lipophilic formulations, likely due to stronger interactions of the latter samples with the intercellular lipid matrix of the SC.

Compared to the semisolids, the liquid ME formulation provided the lowest accumulation of the drug within the skin tissues, despite exhibiting higher drug liberation than LCR and OM when synthetic membranes were used. This implies that complex interactions between topical preparations and skin compartments cannot be reliably predicted by artificial membranes. This is not a rare case, as there have been similar discrepancies documented elsewhere [8,10,34]. However, considering that nanocarriers, such as MEs, have shown great potential in enhancing drug delivery by overcoming biological barriers, including the challenging delivery of nucleic acids for brain disease treatment [50], the observed result was therefore unexpected. In (trans)dermal drug delivery, improved drug penetration by MEs can be ascribed to the several factors, to mention just a few: the concentration gradient across the skin, the presence of chemical penetration enhancers as system constituents (oil, surfactants), drug diffusion through the dermal appendage route (hair follicles and sweat glands) skin hydration and the reservoir effects of the system’s inner phase [7]. A possible explanation for the lower skin concentrations of PRO-HCl obtained with the ME vehicle could be an insignificant driving force for partitioning into the lipophilic SC. This might be due to the drug’s greater affinity and solubility in the ME ingredients, as previously also observed by Kelchen and Brogden [14]. Their study revealed a significantly lower amount of PRO-HCl was obtained from MEs than from a simple drug solution in PBS.

To estimate the systemic absorption of the drug and to either achieve transdermal drug delivery or exclude potential side effects in selective intradermal drug delivery, a permeation study was performed. Under the experimental setup used, no permeation of PRO-HCl through the skin was observed upon topical application of OM, LCR and ME, as no drug was detected in the receptor medium. Therefore, prevention of PRO-HCl entry into systemic circulation from these formulations is anticipated, suggesting that targeted intradermal drug delivery is achievable with the topical application of these three investigated samples. These results were not expected, as similar studies have reported permeation of PRO-HCl across porcine full-thickness skin and epidermis from various formulations administered under either *finite* or *infinite* dosing regimens [8,10,14,35]. However, findings obtained with HG are consistent with these latter works. Namely, a small fraction of the drug applied via HG reached the receptor department only after 24 h (2.95 ± 0.13 µg/cm^2^), while no PRO-HCl was detected in samples acquired prior to that. Although this fraction was more than sixfold lower than the drug amount retained within the skin, it demonstrates the HG’s ability to modulate diffusion and allow partial transdermal transport. The presence of water-rich domains and interconnected pores in the HG matrix likely facilitated this limited but sustained permeation through the hydrated tissue barrier.

Therefore, according to this part of the study, the selective intradermal delivery of PRO-HCl required in infantile hemangiomas treatment can be achieved using either liquid colloidal vehicle (ME) or semisolid formulations. Samples delivering lower doses of the drug into the skin appear promising for the development of topical formulations for newborn infants. On the other hand, with the exception of the HG formulation, none of the investigated samples appeared promising for achieving systemic drug effects in cardiovascular diseases. However, although the skin model used here does not directly represent the biological target tissue of PRO therapy, it should be emphasized that our study was designed as a preliminary evaluation of the formulation’s ability to enhance dermal permeation. Thus, taking into account that the absence of blood vessels in the skin model used for ex vivo permeation studies might lead to an overestimation of drug retention within the skin [51] and the abovementioned constraints, an appropriate in vivo study is necessary to confirm these findings.

### 2.5. Ex Vivo MNs-Assisted Drug Penetration/Permeation Studies

**Figure 7:**
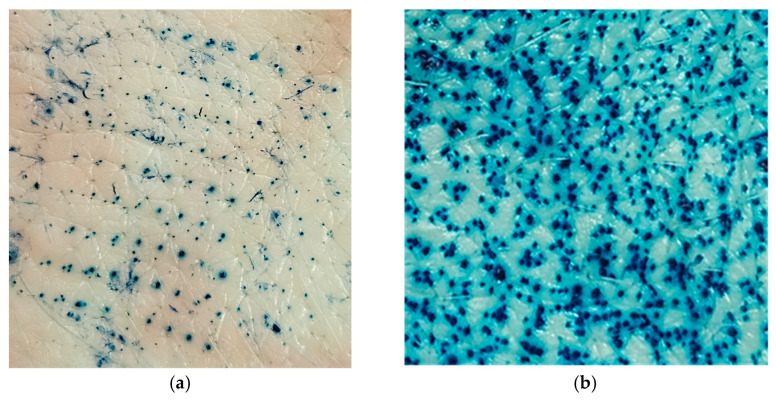
Skin insertion methods for MNs: (**a**) applied with a downward force of 3.8 N; (**b**) manual rolling forward and back five times along a y axis.

In order to investigate the possibility of further enhancing drug transport across the skin and achieving transdermal drug delivery, the formulations exhibiting the highest and lowest ability for drug transport were applied to skin sections pretreated with solid MNs. Since application of MNs to the skin causes formation of water filled microchannels in the outer skin layers, passive diffusion is enabled. Nevertheless, the disruption of skin barrier properties by using this type of penetration enhancer is strongly influenced by the type and characteristics of the MNs, as well as their insertion method [6,52]. As a confirmation of micropore creation in the skin upon MNs application, a dye binding study was performed. As can be seen from Figure 7, both methods of MN insertion in the skin (manual rolling and using a weight) led to micropore creation in the skin, disrupting the SC barrier function. Unlike rolling, which generates shearing forces that disrupt the skin, the static impact insertion of MNs using a weight involves a perpendicular force. Also, increasing the number of unilateral rolls, the extent of skin disruption can be further modulated. Consequently, due to the larger number of microchannels formed and greater permeability achieved by manual rolling, the skin samples used in this experiment were pretreated manually with a MNs roller prior to the application of HG and ME samples.

**Figure 8:**
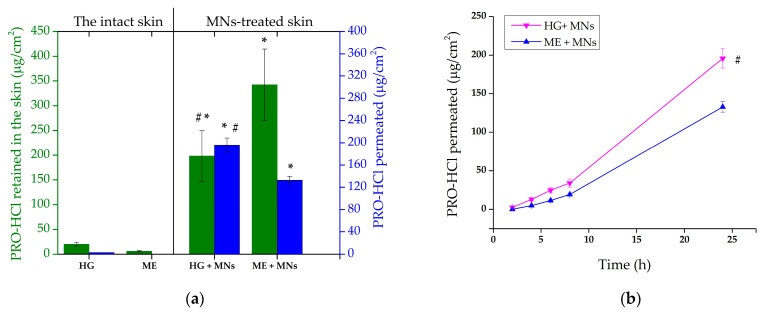
(**a**) Ex vivo penetration/permeation of PRO-HCl from the investigated formulations determined across full-thickness porcine ear skin perforated with MNs under infinite dose conditions (mean ± SD, *n* = 3). (**b**) The permeation profiles of PRO-HCl through full-thickness porcine skin perforated with MNs under *infinite* dose conditions in 24 h (mean ± SD, *n* = 3); * statistically significant change (*p* < 0.05) compared to the results obtained with the intact skin; # statistically significant change (*p* < 0.05) compared to ME + MNs. HG—hydrogel; ME—microemulsion; MNs—microneedles.

As expected, the insertion of solid MNs significantly increased PRO-HCl transport, both into and through the skin (Figure 8, Table 3). The amount of drug retained in the MN-treated skin sections reached 198.19 ± 51.41 µg/cm^2^ and 342.34 ± 72.36 µg/cm^2^ after application of HG and ME, yielding intradermal delivery enhancement ratios of 8.7 and 60.3, respectively. Taking into account the results of the experiment conducted on untreated skin, the superior effect of MNs-assisted cutaneous delivery of PRO-HCl from the ME sample was surprising. The greater ability of the ME formulation to deliver PRO-HCl intradermally could derive from the different rheological characteristics of the samples. Due to the ME’s lower viscosity, its diffusion through the water microchannels formed upon MN insertion would be facilitated. This assumption is supported by studies showing that a higher drug flux via MN-mediated approaches was achieved using less viscous formulations compared to those with higher viscosity [53].

Comparable studies have also demonstrated enhanced drug penetration when MEs were applied in combination with MNs [14,33,54,55]. However, the magnitude of improvement reported for other model drugs, such as celecoxib [55] and sertaconazole nitrate [33], was typically modest (less than a twofold increase compared to MEs alone). In contrast, PRO-HCl exhibited up to a 20-fold rise in skin accumulation following MN pretreatment [14], consistent with the results observed in our work. Such discrepancies among studies are likely attributable to variations in experimental conditions, including MN design, insertion technique, dosing regimen, and the presence of occlusion. In the present study, higher enhancement can reasonably be ascribed to the larger applied dose, occlusive setup, and greater number of micropores created during MN application.

Further confirmation of micropore formation in the skin was provided by PRO-HCl quantified in the receptor medium (Figure 8). To be more precise, the cumulative drug amount permeated from HG after 24 h through the MN-pretreated skin was 195.85 ± 12.77 μg/cm^2^, whereas the ME formulation produced significantly lower permeation under the same conditions (132.76 ± 6.88 μg/cm^2^, *p* = 0.0017). The enhanced drug permeation observed might be attributed to the height of MNs used, which was 500 µm. Due to the fact that the use of longer MNs generally leads to lower skin deposition of a hydrophilic drug and higher transport across the skin, this result was somewhat anticipated [56]. High permeation of PRO-HCl could also be a consequence of the enhancing effect of occlusive conditions employed in our study. For instance, PRO skin retention increased only slightly when polymeric films were applied under occlusion, while drug permeation increment was considerably higher compared to drug transport in a non-occlusive environment [16].

The different results obtained with HG and ME highlight a critical difference in drug disposition profiles between the two delivery systems. Although the ME exhibited greater total skin loading, the HG achieved a more balanced ratio of intradermal retention to transdermal permeation (Table 3). This indicates that the HG matrix maintained sufficient drug availability within the micropores to enable gradual diffusion, while simultaneously ensuring sustained intradermal drug deposition. The hydrated, viscoelastic structure of the HG likely facilitated this dual behavior by preserving local moisture, maintaining intimate skin contact, and providing a diffusion-controlled release environment.

Drug permeation (Figure 8b) from the evaluated formulations could also be described by different kinetic models. When the dialysis membranes were replaced with MN-treated skin, both formulations exhibited significant shifts in their kinetic behavior. For the HG, the mechanism transitioned from Korsmeyer–Peppas to zero-order kinetics (r^2^ = 0.9881), indicating that once microchannels were introduced, the gel functioned as a constant-rate reservoir capable of maintaining a stable flux of dissolved drug through the microporous barrier [57]. This transition implies that the hydrated polymeric matrix compensated for concentration gradients by releasing PRO-HCl in a sustained, concentration-independent manner. The viscoelastic nature and water-holding capacity of the HG likely preserved continuous hydration within the microchannels, maintaining both diffusion and polymer relaxation in dynamic equilibrium. On the other hand, the ME displayed the opposite trend as its kinetic model shifted from Higuchi to Korsmeyer–Peppas (r^2^ = 0.9984) upon MN pretreatment. This change suggests that the structural disruption of the skin barrier altered the diffusion path and introduced elements of non-Fickian transport, possibly related to the ME’s microstructure change, droplet reorganization, interfacial relaxation, and enhanced partitioning within the microchannels. Unlike the HG, the ME system showed higher sensitivity to environmental perturbations, resulting in less predictable release dynamics.

Because the aim of selective intradermal delivery was to reach maximal drug skin concentration without permeation, the relatively high PRO-HCl percutaneous absorption achieved with MN skin pretreatment was not desirable. However, when considering the content of the drug deposited within the skin and the drug permeated through the skin in the receptor medium (skin to receiver ratio), it is obvious that the combined application of MNs and ME favors PRO-HCl intradermal delivery more pronouncedly than the combination of MNs and the HG sample (ratios of 2.6 and 1.0, respectively). Although the difference in the total amount of drug transported into and across the skin (penetration + permeation) was not of a statistical significance for the two tested formulations (*p* = 0.183), the distribution pattern of the drug in the skin tissues and the receptor compartment was quite distinct. According to this, the ME formulation combined with MNs appears to be more appropriate for intradermal PRO-HCl delivery, whereas HG coupled with solid MNs could potentially be used for improving transdermal drug delivery. However, the results of penetration/permeation studies require careful consideration, as there have been instances where drug permeation attained during ex vivo experiments, while drug percutaneous absorption in systemic circulation was not detected in vivo [58]. In support of this, despite the low plasma levels of PRO-HCl measured upon topical application of a gel formulation in rats, Zhou et al. reported good tolerability and no side effects in infants [59].

Taking into account the results obtained using synthetic membranes and porcine skin, the observations regarding the influence of vehicle microstructure on PRO-HCl release and skin disposition, are consistent with recent reports that link HG nanodomain architecture to controlled release and tissue repair efficacy [21,22,23], suggesting that targeted tuning of the HG network may further enhance both local therapeutic concentrations and regenerative outcomes.

Finally, before the final conclusions can be drawn, additional research is necessary. Namely, to gain better insight and identify the most significant parameters influencing drug delivery into/through the skin, a deeper understanding of the internal structure of the tested formulations is required. Furthermore, while the experimental conditions used for performing the ex vivo penetration/permeation test represent a standard setup in this type of studies [35,58,60,61], it is important to consider that the experimental setting (e.g., skin type, study duration, *infinite* and/or *finite* dose regimen) can affect the fate of a topical drug. Therefore, further investigations using disease-relevant in vitro models that mimic a real dosing regimen in a clinical setting (*finite* dosing and non-occlusive conditions), thus, better reflecting the in vivo situation, along with the histological study should be carried out.

## 3. Conclusions

In summary, this study provides the first systematic comparison of HG with commonly compounded topical vehicles for PRO-HCl under identical conditions and uniquely demonstrates how vehicle selection, with or without MN pretreatment, can be tailored to either local intradermal therapy of infantile hemangiomas or systemic transdermal delivery for cardiovascular indications. HG demonstrated the highest drug retention within the skin, but this was accompanied with low drug permeation. Comparisons with other vehicles (OM, LCR, and ME) revealed that factors such as water content, pH, and rheological properties strongly influence drug transport. While ME showed the greatest potential for enhancing PRO-HCl penetration when combined with MNs, HG exhibited the greatest potential for PRO-HCl systemic exposure. These findings underscore the relevance of HG as a practical and effective vehicle for intradermal PRO-HCl administration, while its combination with MNs could be considered as a mean to achieve systemic effects of PRO-HCl. Therefore, this study provides a foundation for further optimization and in vivo evaluation to fine-tune topical and transdermal drug delivery strategies. However, although pharmacopeial HGs have an established safety profile, future studies should include dedicated biocompatibility assessments (e.g., skin irritation or sensitization assays) to further substantiate safety for extended or repeated application. In addition, a deeper understanding of the inner microstructure and the mechanisms of PRO-HCl skin diffusion must also be provided.

## 4. Materials and Methods

### 4.1. Materials

PRO in the form of hydrochloride salt (PRO-HCl) was purchased from TCI EUROPE N.V, Belgium. Lanolin and carmellose sodium were obtained from Semikem, Sarajevo, Bosnia and Herzegovina, whereas liquid paraffine and petrolatum were purchased from Fagron (Zagreb, Croatia). Ethanol was supplied by Gram-Mol (Zagreb, Croatia). Glycerol was obtained from Lach-Ner (Neratovice, Czech Republic) and polysorbate 80 from Sigma–Aldrich Laborchemikalien GmbH (Seelze, Germany). Propylene glycol monocaprylate (Capryol^TM^ 90) was kindly gifted from Gattefosse (Lyon, France). Ready-to-use vehicle Pentravan^®^ was obtained from Fagron (Rotterdam, The Netherlands). Pentravan^®^ represents oil-in-water semisolid emulsion system composed of carbomer 940 and lecithin, forming the polymeric and liposomal matrix, respectively. The other components include carbamide, dimethicone, butylhydroxytoluene, potassium sorbate, cetyl alcohol, stearyl alcohol, polyoxyethylene stearate, stearic acid, glycerol monostearate, benzoic acid and hydrochloric acid. Double-distilled water was obtained with a Barnstead^TM^ LabTower^TM^ EDI Water Purification System (Thermo Scientific^TM^, Waltham, MA, USA). All other chemicals were of pharmaceutical grade and used as received without purification.

### 4.2. Preparation of Formulations

In the current study, four different formulations were used as vehicles for incorporation of PRO-HCl (1%, *w*/*w*): carmellose HG, hydrophilic petrolatum OM, commercially available LCR (oil-in-water) base Pentravan^®^ and ME (Table 4). Hydrophilic petrolatum OM and carmellose HG, correspond to official pharmacopeial formulations (Magistral formulae 2008 of the Republic of Serbia) commonly used in magistral topical preparations. Their composition and quality comply with pharmacopeial standards, which ensures established biocompatibility and suitability for dermal application. PRO-HCl concentration of 1% *w*/*w* was selected based on previous studies indicating that this range is effective while minimizing the risk of local side effects [4,10,35].

HG formulation was prepared by firstly dissolving PRO-HCl in freshly boiled and cooled purified water. This solution was then used for dispersing carmellose sodium 600 by gradual addition of the solution to the gelling agent. The resulting mixture is then left to swell and after complete swelling (approximately 2 h), glycerol (85%) was added with gentle stirring and the mixing was continued until a homogeneous gel is formed.

For the preparation of OM, ingredients (Table 4) were transferred to a mortar, heated to 70 °C using water bath and melted during gentle mixing. Then, the mixture was cooled at room temperature using stirring. In order to minimize discrepancies in viscosity, particle size and air inclusion, PRO-HCl (1%, *w*/*w*) was carefully suspended in the prepared vehicle under controlled conditions. Hence, the vehicle was gradually added to the drug previously levigated with few drops of liquid paraffin until homogenous dispersion was obtained.

To prepare LCR, PRO-HCl (1%, *w*/*w*) was transferred to a porcelain mortar and gently stirred after adding a small amount of water (2 drops), then the appropriate amount of Pentravan^®^ (up to 100.0 g) was added step by step avoiding the air inclusion, until a homogenous dispersion was obtained.

Finally, as a ME vehicle, previously developed biocompatible ME selected from the constructed pseudo-ternary phase diagram (Supplementary Materials Figure S1) was employed and prepared as explained elsewhere [62]. In brief, first the mixture of surfactant (polysorbate 80) and cosurfactant (ethanol) (1:1, *w*/*w*) was prepared using a magnetic stirrer for 15 min at 300 rpm (IKA^®^ KS 260 basic shaker, IKA-Werke GmbH & Co. KG, Staufen, Germany). Then, the oil phase (Capryol^TM^ 90) was added to this mixture and stirred at the same rate for another 15 min. Afterwards, the proper amount of double-distilled water (60%, *w*/*w*) was added precisely dropwise with gentle stirring for additional 30 min at 300 rpm until homogenous, transparent, non-opalescent and low-viscous ME sample was obtained. ME loaded with PRO-HCl was prepared by dissolving the drug (1%, *w*/*w*) in previously prepared ME formulation with stirring on a magnetic stirrer for 2 h also at mixing rate of 300 rpm. In order to ensure the complete drug solubilization, the sample was subjected to ultrasonication for 15 min at room temperature (Sonorex Digitec DT 102H, Bandelin, Berlin, Germany).

### 4.3. Characterization of the Investigated Formulations

PRO-HCl loaded topical formulations were subjected to assessment of their visual appearance, light optical microscopy (where applicable), pH measurements (where applicable), electrical conductivity studies (where applicable), droplet size measurements, rheological analysis and drug content.

The microscopic images of HG, OM, and LCR were performed with Leica DM 6000B optical microscope (Leica, Wetzlar, Germany) equipped with Leica DFC 310FX camera. The images were captured under a magnification of 50× and analyzed using LAS V4.12 software.

pH values were measured by a direct immersion of the pH meter into HG, LCR and ME samples (pH meter 3110, WTW, Weilheim, Germany). Experiments were conducted in triplicate for each sample.

In order to obtain information about the structure of ME sample, electrical conductivity was evaluated with conductometer (Multi 9620 IDS, WTW, Weilheim, Germany). Mean droplet size (Z-ave) and polydispersity index (PDI) of ME were analyzed by photon correlation spectroscopy (PCS) at 20 °C, using Zetasizer Nano ZS (Malvern Instruments Ltd., Worcestershire, UK). The sample was not diluted for this purpose in order to prevent a structural change upon dilution. Zeta potential was not determined as it is not routinely applied to thermodynamically stable MEs [29]. Also, none of the other formulations contained discrete colloidal particles (HG and LCR: molecular dispersion of PRO-HCl; OM: non-colloidal drug aggregates), thus, measurements of zeta potential were not applicable.

Rheological behavior of all samples was carried out by HAAKE MARS rheometer (Thermo Scientific, Karlsruhe, Germany) at 25 ± 0.1 °C.

The flow curves of ME were determined using cone-plate C35 2°/Ti sensor. The samples were exposed to continual increase in shear rate from 0.001 to 100 s^−1^ during 45 s. Then, the shear rate was kept constant at 100 s^−1^ for 30 s and finally, during the next 45 s, it was decreased to 0 s^−1^.

For HG, OM, and LCR oscillatory rheological measurements were performed for using plate-plate P35/Ti sensor. Amplitude sweep tests were performed in order to determine linear viscoelastic region (LVR). Storage modulus G’ and viscous modulus G” were recorded versus shear stress (1–1000 Pa) at constant frequency of 1 Hz. The appropriate shear stress was chosen as middle of LVR from plot of G’ and G” versus shear stress. This shear stress was applied in frequency sweep tests, where G’ and G” moduli were recorded versus frequency (0.1–10 Hz) at constant shear stress.

An assessment of the investigated semisolid samples regarding the drug content uniformity was conducted in accordance with the method described in the United States Pharmacopeia (USP) for products packaged in jars [63]. A syringe barrel (without the plunger), with its bottom end removed, was inserted vertically into the container containing the test sample until it reached the bottom. It was then gently twisted to collect a core sample. The syringe barrel was carefully removed, and the plunger was inserted to extrude the sample, which represented the top, middle, and bottom zones of the jar. Precisely weighed portions (500 mg) from each zone were transferred into a flask containing 10 mL of the extraction solvent (methanol/water mixture, 30% *v*/*v*). The mixture was than vortexed and afterwards sonicated for 5 min (Sonorex RK 120H, Bandelin, Berlin, Germany). Finally, it was centrifuged for 15 min at 30,000 rpm (Centrifuge MPW-56; MPW Med. Instruments, Warszawa, Poland) and filtered through 0.45 µm membrane filters. Before the samples were analyzed using high performance liquid chromatography (HPLC), they were diluted 10-fold with methanolic water (30%, *v*/*v*) [10]. The same procedure was carried out with ME, with the only difference being sample collection with a nondamaged syringe barrel with the plunger.

### 4.4. In Vitro Drug Release Studies

In vitro drug release study was performed on Franz-type vertical diffusion cells (PermeGear, Hellertown, PA, USA) according to the USP general monograph for semisolid drug products [64]. Receptor chambers with a volume of 12 mL were filled with degassed preheated (32 °C) receptor fluid (PBS pH 7.4) and the investigated samples (500 mg) were uniformly applied on the surface of previously activated cellulose dialysis membranes (pore size 2.4 nm and molecular weight cut-off 12,000) (Sigma Aldrich, Saint Louis, MO, USA), which were carefully mounted between the donor and receptor compartments. The effective diffusion area was 2.0 cm^2^. Hence, an *infinite* dose regimen is used, applying larger amounts to minimize depletion of the active substance due to evaporation or diffusion, and allowing for parametric characterization of drug release and comparison of formulations. In order to prevent any evaporation during the experiments, the donor chamber was covered with occlusive Parafilm^TM^ M (Bemis, Neenah, WI, USA). Stirring speed was 500 rpm and the temperature of diffusion cells was kept at 32 °C during the entire experiment. The saturation solubility of PRO-HCl was determined as 70.19 ± 4.91 mg/mL (*n* = 3), hence, *sink* conditions were provided. At predetermined time intervals (1 h, 2 h, 4 h, 6 h, 8 h and 24 h upon application) appropriate samples (600 µL) were collected and immediately replaced with the same volume of fresh pre-heated receptor medium as to maintain *sink* conditions. The acquired samples were then analyzed for PRO-HCl using HPLC method. The obtained results were presented as the cumulative amount of drug released per unit area (μg/cm^2^) plotted versus time (t), which enabled the calculation of in vitro release rate for each tested sample. To compare the obtained drug release profiles, model-dependent and statistical analyses were proposed. Mathematical models utilized for the investigation of kinetic release of PRO-HCl from each sample and the rationalization of the mechanisms involved in this process were: zero-order, first-order, Higuchi and Kormeyer-Peppas. The values of the correlation coefficient for each model (r^2^) were used as a criterion for determination of the most suitable kinetic model.

### 4.5. Ex Vivo Drug Penetration/Permeation Studies

In order to assess penetration and permeation of PRO-HCl from the investigated formulations, a set of ex vivo drug penetration/permeation studies was carried out using full-thickness pig ear skin mounted on Franz diffusion cells. Porcine skin was supplied from the local abattoir directly after animals slaughtering. Pig ears were washed with cold tap water immediately after that. Then, they were dried and frozen at −20 °C (maximum to 30 days). On the day of the study, porcine ears were thawed at room temperature, and any visible hair was removed using an electric hair clipper. A surgical blade was used in order to remove subcutaneous fat tissue. The skin prepared in this way was punched into suitable sections with a diameter of 25 mm and inspected visually with a magnifier to ensure there was no sign of physical damage. The obtained skin sections were then rehydrated in the receptor fluid during 1 h and cautiously clamped between the donor and receptor parts of diffusion cells positioning dermis layer to face the receptor compartment. Experimental setup and the analysis of the obtained data were the same as for the in vitro release test (4.4. In vitro drug release studies) except that samples were collected from the receptor medium after 2 h, 4 h, 6 h, 8 h, and 24 h upon application of the formulations. When the penetration/permeation test was finished, skin samples were analyzed for PRO-HCl content using the HPLC method. Before quantitative analysis, in order to eliminate excess of formulations, skin sections removed from the Franz cells were wiped clean three times using PBS 7.4. Afterwards, they were cut into smaller pieces and transferred into glass tubes filled with 5 mL of methanol [6,14] and then subjected to homogenization using a homogenizer at 850 rpm for 5 min (HG-15D homogenizer, Witeg Labortechnik GmbH, Wertheim, Germany). In order to achieve effective extraction of the drug, the samples were then shake on a laboratory shaker (IKA^®^ KS 260 basic shaker, IKA-Werke GmbH & Co. KG, Staufen, Germany) and sonicated (Sonorex RK 120H, Bandelin, Berlin, Germany) twice for 30 min. After that centrifugation was performed for 20 min at 4000 rpm (Centrifuge MPW-56; MPW Med. Instruments, Warszawa, Poland). Finally, the obtained supernatants were filtered through membrane filters (0.45 µm), and subjected to quantitative analysis.

### 4.6. Ex Vivo Drug Penetration/Permeation Studies on MNs Preterated Skin

**Figure 9:**
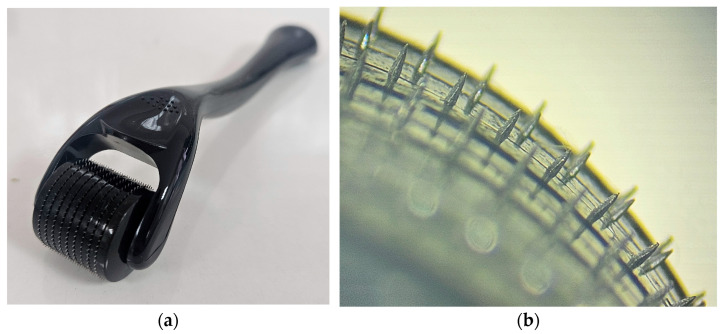
(**a**) The MN roller and MN head arrangement; (**b**) representative micrographs of MNs geometry using light microscopy.

For skin drug retention and permeation after the application of selected samples on skin pretreated with solid titanium MNs, the same experimental procedure was followed as for intact skin. The only difference was in the preparation of the skin samples, which required piercing the skin sections for the MNs studies. Namely, skin diffusion area was treated with solid titanium MNs roller (Derma Roller, Dr. Pen, Guangzhou, China). MNs height was 500 µm and the roller had 540 needles arranged as nine MNs in 60 rows (Figure 9). Before the application of formulations, to perforate the skin, the roller was placed onto the skin and manually rolled forward and back five times along a single axis. Since the same operator applied the same speed, angle, pressure of application and coverage each time, the reproducibility of MN application across all samples was provided. As part of this study, preliminary ex vivo skin insertion test was performed to assess the penetration ability of the MNs used. Different application techniques were compared as to select the most appropriate method for achieving porcine skin perforation. To perforate the skin samples, the roller was either placed onto pig ear skin clamped on supporting Styrofoam and manually rolled back and forward five times along a single axis (y) or a 500 g weight (equivalent to a force 3.8 N) was attached to the roller for 30 s [65]. Then, a dye binding study was conducted. In brief, on MNs pretreated skin, a solution of methylene blue was applied in excess and leakage of the dye was prevented with a metal ring. Three minutes later, the excess dye was wiped from the skin sections using ethanol. Considering that methylene blue is hydrophilic, it cannot be absorbed by the SC but can bound to the proteins of the viable skin tissue. Therefore, if the SC is perforated by MNs, the dye will diffuse across the horny layer and bind to proteins within deeper skin layers [33].

### 4.7. HPLC Analysis

In order to quantify the drug released from the samples, as well as PRO-HCl diffused into and across the porcine ear skin, a validated HPLC method was utilized [66]. The drug was separated on Agilent 1200 series system (Agilent Technologies, Santa Clara, CA, USA) equipped with a quaternary pump (G1311A) and an autosampler (ALS G1329A). The analytical column selected was Zorbax Eclipse C18 (150 × 4.6 mm, 5 μm particle size). The mobile phase consisted of a mixture of 50 mM phosphate buffer (pH adjusted to 5.5 with orthophosphoric acid) and acetonitrile (60:40, *v*/*v*). The flow rate was 1 mL/min, and the column temperature was maintained at 25 °C. The injection volume was 10 µL. The UV detection was performed using a DAD detector at 292 nm.

### 4.8. Statistical Analysis

Wherever applicable, the obtained data were presented as mean ± standard deviation (SD). Statistical analysis was performed using either Student’s *t*-test or one-way analysis of variance (ANOVA), depending on the number of groups analyzed. Statistical significance was set at *p* < 0.05. For data analysis, the GraphPad Prism version 7 software package for Windows (GraphPad Software Inc., La Jolla, CA, USA) was employed.

## Figures and Tables

**Table 1:** pH values and drug content of the investigated formulations.

Formulation Code	pH (Mean ± SD, *n* = 3)	Drug Content (%) (Mean ± SD, *n* = 3)	Drug Content (%) (Mean ± SD, *n* = 3) After 1 Month
HG	7.08 ± 0.02	97.76 ± 3.12	95.96 ± 2.78
OM	6.19 ± 0.11	86.69 ± 1.84	84.17 ± 1.45
LCR	5.19 ± 0.07	95.27 ± 1.21	96.42 ± 4.36
ME	5.44 ± 0.04	100.24 ± 2.48	98.69 ± 1.93

**Table 2:** In vitro release parameters of formulations loaded with 1.0% (*w*/*w*) of PRO-HCl obtained across the synthetic cellulose dialysis membrane (mean ± SD, *n* = 3).

Formulation Code	Release Rate (µg/cm^2^h^−1/2^)	Q_24h_ (µg/cm^2^)	PRO-HCl Released (%)	Kinetic Model (r^2^)
HG	492.97 ± 12.81 ^a^	2396.86 ± 59.80 ^a^	48.18 ± 1.20 ^a^	Korsmeyer-Peppas (0.998)
OM	10.68 ± 1.02 ^b^	50.41 ± 3.87 ^b^	1.01 ± 0.08 ^b^	Higuchi (0.9722)
LCR	22.08 ± 0.26 ^b^	106.53 ± 0.76 ^b^	2.14 ± 0.01 ^b^	Higuchi (0.9996)
ME	300.40 ± 7.77 ^a^	1395.47 ± 33.99 ^a^	28.05 ± 0.68 ^a^	Higuchi (0.9995)

^a^ *p* < 0.05 compared to all other samples, ^b^ *p* < 0.05 compared to HG and ME.

**Table 3:** Ex vivo permeation/penetration parameters of formulations loaded with 1.0% (*w*/*w*) of PRO-HCl obtained across the intact and MN-treated full-thickness porcine skin (mean ± SD, *n* = 3).

Formulation Code	PRO-HCl Penetrated (Intact Skin) (µg/cm^2^)	PRO-HCl Penetrated (MN-Treated Skin) (µg/cm^2^)	Enhancement Ratio	Permeation Rate (MN-Treated Skin) (µg/cm^2^h)	PRO-HCl Permeated (MN-Treated Skin) (µg/cm^2^)
HG	19.87 ± 4.12 ^a^	198.19 ± 51.41 ^a^	8.7	9.07 ± 3.39	195.85 ± 12.77 ^a^
ME	5.85 ± 1.34	342.34 ± 72.36	60.3	6.30 ± 1.98	132.76 ± 6.88

^a^ *p* < 0.05 compared to ME.

**Table 4:** Qualitative and quantitative composition of the selected formulations (%, *w*/*w*) as vehicles for incorporation of PRO-HCl at a concentration of 1% (*w*/*w*).

Ingredients	Formulation Code			
	HG	HG	LCR	ME
Carmellose sodium 600	5.0	-	-	-
Glycerol, 85%	10.0	-	-	-
Cholesterol	-	5.0	-	-
Lanolin	-	15.0	-	-
Liquid paraffine	-	15.0	-	-
Petrolatum	-	65.0	-	-
Pentravan^®^	-	-	up to 100.0	-
Capryol^TM^ 90	-	-	-	8.0
Polysorbate 80	-	-	-	16.0
Ethanol	-	-	-	16.0
Double-distilled water	85.0	-	q.s.	60.0

## Data Availability

The data presented in this study are available in this article.

## Supplementary Materials

The following supporting information can be downloaded at: https://www.mdpi.com/article/10.3390/gels12010010/s1, Table S1. Saturation solubility of PRO-HCl in microemulsion ingredients; Figure S1. Pseudo-ternary phase diagrams of system containing Capryol^TM^ 90, Polysorbate 80-ethanol (1:1, *w*/*w*) and water (the green region represents monophasic microemulsion area with the marked position of the selected formulation).

## Abbreviations

The following abbreviations are used in this manuscript:

PRO-HCl	Propranolol hydrochloride
SC	The stratum corneum
OM	Ointment
LCR	Liposomal cream
HG	Hydrogel
ME	Microemulsion
MNs	Microneedles
