# Supplementary material for: Hydrogel Versus Alternative Vehicles for (Trans)dermal Delivery of Propranolol Hydrochloride—In Vitro and Ex Vivo Studies
Source: Gels. 2025 Dec 23;12(1):10. doi: 10.3390/gels12010010 (PMC12840661; doi:10.3390/gels12010010)
Supplement: Supplementary file 1 [file gels-12-00010-s001.zip › gels-4024215-supplementary.pdf]

Supplementary Material

Hydrogel Versus Alternative Vehicles for (Trans)dermal Delivery of Propranolol Hydrochloride—In Vitro and Ex Vivo Studies

Nataša Bubić Pajić[1*], Milica Kaurin[2], Adrijana Klepić[3], Darija Knežević Ratković[1], Aneta Stojmenovski[4], Veljko Krstonošić[5] and Ranko Škrbić[6,7,8]

1 Department of Pharmacy, Faculty of Medicine, University of Banja Luka, Save Mrkalja 14, 78000 Banja Luka, Bosnia and Herzegovina; darija.knezevic.ratkovic@med.unibl.org
2 Berlin-Chemie Menarini AG, Hasana Brkica 2/II, 71000 Sarajevo, Bosnia and Herzegovina; kaurinmilica@gmail.com
3 ZU Apoteke "B Pharm", Kulska Obala Bb, 79220 Novi Grad, Bosnia and Herzegovina; klepicadrijana@yahoo.com
4 Centre for Biomedical Research, Faculty of Medicine, University of Banja Luka, Save Mrkalja 16,
78000 Banja Luka, Bosnia and Herzegovina; aneta.stojmenovski@med.unibl.org
5 Department of Pharmacy, Faculty of Medicine, University of Novi Sad, Hajduk Veljkova 3, 21000 Novi Sad, Serbia; veljko.krstonosic@mf.uns.ac.rs
6 Department of Pharmacology, Toxicology and Clinical Pharmacology, Faculty of Medicine, University of Banja Luka, Save Mrkalja 16, 78000 Banja Luka, Bosnia and Herzegovina; ranko.skrbic@med.unibl.org
7 Academy of Sciences and Arts of the Republic of Srpska, Bana dr Todora Lazarevića 1, 78000 Banja Luka, Bosnia and Herzegovina
8 Department of Pathologic Physiology, I.M. Sechenov First Moscow State Medical University,
Moscow 119435, Russia
* Correspondence: natasa.bubic.pajic@med.unibl.org

S2. Results and Discussion

S2.1. Preparation of Formulations

Table S1. Saturation solubility of propranolol hydrochloride in microemulsion ingredients

Excipient	Solubility (mg/mL ± SD, *n* = 3)
Double distilled water	88.1 ± 5.4
Ethanol	47.5 ± 2.6
Capryol™ 90	0.6 ± 0.1
Polysorbate 80	15.6 ± 1.3

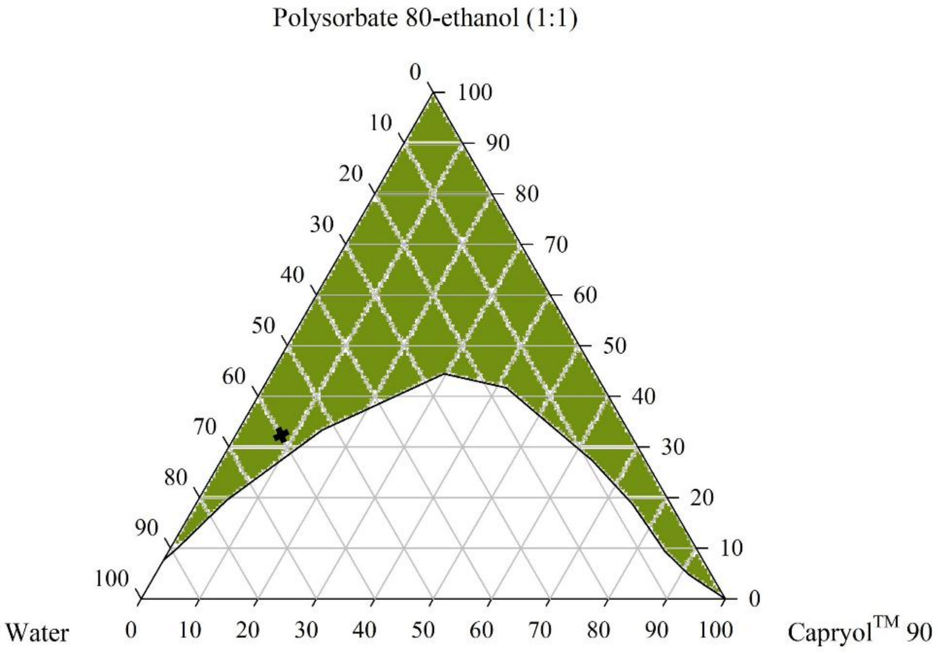

Figure S1. Pseudo-ternary phase diagrams of system containing Capryol™ 90, Polysorbate 80-ethanol (1:1, w/w) and water (Green region represents monophasic microemulsion area with marked position of the selected formulation)

S4. Materials and Methods

S4.2. Preparation of Formulations

Construction of Pseudo-ternary Phase Diagram

Phase behavior assessment of systems comprising of oil (Capryol™ 90), mixture of surfactant and cosurfactant (polysorbate 80 and ethanol) and water was conducted by construction of pseudo-ternary phase diagrams, using water titration method. The mixture of surfactant and cosurfactant at a ratio of 1:1 (*w/w*) and oil were mixed in weight ratios ranging from 9:1 to 1:9 (*w/w*), then water was gradually added dropwise (Vortex Mixer, Velp Scientifica, Usmate Velate, Italy). The boundary of the microemulsion region was defined as the point at which the maximum amount of water was incorporated into homogeneous, low-viscous, transparent, and non-opalescent microemulsion samples. Systems that became opalescent or turbid were considered indicative of phase separation.

Solubility Study of Propranolol Hydrochloride

The solubility studies of propranolol hydrochloride in the water phase, oil (Capryol ™ 90), surfactant (polysorbate 80) and cosurfactant (ethanol) were performed by shake flask method utilizing laboratory shaker IKA® KS 260 basic (IKA® Werke GmbH & Co. KG, Staufen, Germany). An excess amount of the drug was added to 5 mL of each excipient in Erlenmeyer flasks and the obtained mixtures were

shaken continuously for 48 h, at a mixing rate of 300 rpm. After that, as to remove the fraction of undissolved drug, the samples were centrifuged for 30 minutes at 3000 min^{-1} (Centrifuge Rotofix 32 A, Hettich, Tuttlingen, Germany). The aliquots of supernatant were then filtered through a membrane filter (0.22 μm). Concentration of propranolol hydrochloride in the supernatant was determined using validated HPLC method.
